# The impact of *KRAS* mutations on VEGF-A production and tumour vascular network

**DOI:** 10.1186/1471-2407-13-125

**Published:** 2013-03-18

**Authors:** Agnès Figueras, Maria Antonia Arbos, Maria Teresa Quiles, Francesc Viñals, Josep Ramón Germà, Gabriel Capellà

**Affiliations:** 1 Translational Research Laboratory, Institut Català d’Oncologia-IDIBELL, Gran Via 199-203, 08908 L’Hospitalet del Llobregat, Barcelona, Spain; 2 Institut de Recerca Vall d’Hebron, Hospital Universitari Vall d’Hebron, Passeig de la Vall d’Hebron 119-129, Barcelona, 08035, Spain; 3 Unitat de Bioquímica i Biologia Molecular; Departament de Ciències Fisiològiques II, Universitat de Barcelona-IDIBELL, Feixa Llarga s/n, 08908 L’Hospitalet del Llobregat, Barcelona, Spain; 4 Department of Medical Oncology, Institut Català d’Oncologia, Gran Via 199-203, 08908 L’Hospitalet del Llobregat, Barcelona, Spain

**Keywords:** *KRAS* mutations, HIF-1α, Vascular endothelial growth factor A, VEGF-A promoter, Tumour angiogenesis

## Abstract

**Background:**

The malignant potential of tumour cells may be influenced by the molecular nature of *KRAS* mutations being codon 13 mutations less aggressive than codon 12 ones. Their metabolic profile is also different, with an increased anaerobic glycolytic metabolism in cells harbouring codon 12 *KRAS* mutations compared with cells containing codon 13 mutations. We hypothesized that this distinct metabolic behaviour could be associated with different HIF-1α expression and a distinct angiogenic profile.

**Methods:**

Codon13 *KRAS* mutation (ASP13) or codon12 *KRAS* mutation (CYS12) NIH3T3 transfectants were analyzed in vitro and in vivo. Expression of HIF-1α, and VEGF-A was studied at RNA and protein levels. Regulation of VEGF-A promoter activity was assessed by means of luciferase assays using different plasmid constructs. Vascular network was assessed in tumors growing after subcutaneous inoculation. Non parametric statistics were used for analysis of results.

**Results:**

Our results show that in normoxic conditions ASP13 transfectants exhibited less HIF-1α protein levels and activity than CYS12. In contrast, codon 13 transfectants exhibited higher *VEGF-A* mRNA and protein levels and enhanced *VEGF-A* promoter activity. These differences were due to a differential activation of Sp1/AP2 transcription elements of the *VEGF-A* promoter associated with increased ERKs signalling in ASP13 transfectants. Subcutaneous CYS12 tumours expressed less VEGF-A and showed a higher microvessel density (MVD) than ASP13 tumours. In contrast, prominent vessels were only observed in the latter.

**Conclusion:**

Subtle changes in the molecular nature of *KRAS* oncogene activating mutations occurring in tumour cells have a major impact on the vascular strategy devised providing with new insights on the role of *KRAS* mutations on angiogenesis.

## Background

Ras proteins have been the subject of intense research as signalling molecules in normal and neoplastic cells [1]. Yet, a complete understanding of their exact mode of action is still to come. Among the three *RAS* genes (*H-RAS*, *KRAS* and *N-RAS*) *KRAS* is the most commonly activated in human tumours. Several lines of evidence suggest that not only the presence or absence of a *KRAS* mutation but its molecular nature influences tumour cell behaviour [2,3]. A reduced transforming capacity of codon 13 mutation as compared with codon 12 is observed *in vitro* and *in vivo*, with short latency times to tumour-appearance for codon 12 *KRAS* overexpressing cells [4-6]. Moreover, our previous results indicate that distinct mutations associate with specific metabolic phenotypes, an increased anaerobic glycolytic metabolism in cells containing codon 12 *KRAS* compared with cells containing codon 13 mutations. Switching to a glycolytic metabolism is a rapid adaptation to hypoxia that can be related to HIF1α expression [7].

Perpetual blood vessel formation and remodelling (angiogenesis) is a hallmark of cancer and a prerequisite for three-dimensional tumour growth, invasion, and metastasis [8]. Hypoxia, by inducing HIF-1α, promotes the expression of VEGF-A, the main pro-angiogenic hypoxia-induced gene [9]. However, oncogenes are also *per se* potent inductors of angiogenesis [10]. Ras proteins are a paradigm for oncogene-dependent induction of tumour angiogenesis due to their involvement in the regulation of key pro and anti angiogenic factors [11-14]. However, its cross-talk with hypoxia-dependent signals is not so clear.

To gain further insight into the metabolic potential and distinct aggressiveness of different activating *KRAS* mutations, we examined the expression levels of HIF-1α and VEGF-A in stable mutated 12 and 13 NIH3T3 transfectants. Our results *in vivo* and *in vitro* indicate that the distinct *KRAS* mutations generated different normoxic HIF-1α responses. Moreover, different VEGF-A expression patterns were observed that are independent of the HIF-1α status but dependent upon ERKs stimulation. These alterations associated with distinct tumoral angiogenic profiles.

## Methods

### Transfectants procedures

#### Generation of transfectants

NIH3T3 cells were produced as previously described [4,15], with plasmid DNA containing a *KRAS* minigene with a G:C A:T mutation (CYS12) at the first position of codon 12 (pMLK12), a G:C A:T mutation (ASP13) at the second position of codon 13 (pMLK13), and a control plasmid containing the expression vector alone (pMLneo). pMLK12, pMLK13, and pMLKwt plasmids were a gift of Dr. Manuel Perucho of the Burnham Institute at La Jolla, CA. Levels of expression of the KRAS protein in the selected clones used were similar [15].

#### Cell culture

Clones were cultured in DMEM supplemented with 20% Fetal Calf Serum and 500 μg/ml of neomycin G418. Mutations were verified by direct sequencing prior to the initiation of every experiment.

#### Inhibitors incubation

Transfected cells cultured 12 hours in FCS deprivation were incubated 15 minutes with the corresponding kinase inhibitor maintaining FCS deprivation. PI3K inhibitor LY294002 (15 μM), p44/42 ERKs inhibitors PD98859 (0.06 mM) or U0126 (20 μM) were obtain by Calbiochem, Ca. Afterwards, next fifteen minutes cells were in contact with FBS and without inhibitors. At the end of incubations, transfected cells were removed from the dishes and we obtained proteins or mRNA as convinced.

### Tumour model

Athymic male nu/nu Swiss mice (Charles River Laboratory, Sta Perpetua, Spain) were injected subcutaneously (s.c.) as previously described [4], according to the protocols approved by the Institutional Animal Care and Use Committee. Tumours were measured periodically with a calliper, and the volume was calculated as length × width^2^ × 1/2 [16]. Tumours were surgically removed and analysed when they reached a diameter of ~ 1 cm.

### Protein expression analysis

#### Western blotting

Cells and tissue samples were lysed with RIPA buffer (1% SDS 10%, 1% NP40, 0.5% de Sodium deoxycholate) plus protease inhibitors. Forty μg of each protein sample were subjected to 10% SDS/PAGE under reducing conditions, and transferred to polyvinylidene fluoride membranes (PVDF-Bio-Rad, Hercules, CA). Membranes were blocked in TBST buffer (0.9% NaCl, 0.02 M Tris (pH 7.5), 0.05% Tween 20; 5% skimmed milk; 1 h, RT), and probed with primary antibodies: anti-pan-Ras (Oncogene Research Products San Diego CA mouse monoclonal clone RAS10 cat: OP40), anti-HIF-1α (a generous gift from Dr. Edurne Berra, CICBiogune, Bilbao, Spain), anti-GLUT-1 (Abcam, Cambridge, UK, ref. Ab652), anti-VEGF-A (Neomarkers, CA, Ref. MS-35), anti-Sp1 (Santa Cruz, CA, ref. SC-59), anti-p-ERKs (Sigma-Aldrich Inc. Monoclonal Anti-Map Kinase activated clone MAPK-YT), anti-p-Akt (Cell Signaling Rabbit Phospho-Akt (Ser473) antibody #9271) and anti-Tubulin (Sigma-Aldrich Inc). Detection was performed using peroxidase-conjugated secondary antibodies. The resulting complexes were visualized by enhanced chemiluminiscence autoradiography (Amersham Life Science,Chicago,Il). Autoradiographs were quantified by scanning densitometry Quantity One Quantitation Software™ (Bio-Rad, Hercules, CA).

#### Enzyme linked immunosorbent assay/ELISA

Expression levels of culture medium cells and tissue associated VEGF were also examined by enzyme linked immunosorbent assay (ELISA: Quantikine immunoassay kits; R&D Systems) according to the manufacturer’s instructions.

#### Vegf Immunohistochemistry

It was performed on paraffin-embedded tissues with VEGF (C-1) mouse monoclonal antibody (Santa Cruz Biotechnology Cat#sc-7269). We used anti-mouse DakoCytomation EnVision System HRP to visualize the reaction.

### RNA expression

#### Total RNA extraction and RT-PCR

Trizol Reagent (Life Technologies Gibco) according to manufacturer’s instructions was used to total mRNA extraction. One μg of RNA was reverse-transcribed into cDNA using pdN6 primers using High Capacity Reverse Transcriptase (Applied Biosystems, Foster city CA). Subsequent Real-Time PCR reaction for *Vegf-A* mRNA levels was performed *in duplo* in the LightCycler^®^ System SYBGreen480 (Roche) using primers: Rev: CACCTTCATGGGACTTCTGCT; Fwd: GCACTGGACCCTGGCTTTA. *Angpt2* mRNA levels were assessed in duplo using inventoried TAQMAN gene expression assays Mm00545822 (Applied Biosystem, Foster city CA). Every gene expression quantification was corrected using three housekeeping genes: mitochondrial ribo-somal protein L19 Mn00452754; Hypoxanthine guanine phosphoribonyl transferase 1 Mn01545399 and Pepti-dylpropyl isomerase A Mn02342430. Threshold cycle data were analyzed using the following formula:  *ratio* = ((*E*_*target*_)^*ΔCP target* (*control*–*sample*)^) [17].

#### Actinomycin D assay

Cellular clones were cultured in 12 well plates and incubated 15, 30 and 180 minutes with Actinomycin D (5 μg/ml) just before RNA total extraction was performed using Trizol Reagent (Life Technologies Gibco) and following manufacturer’s protocol.

### *VEGF* promoter plasmid transfections and luciferase determination

NIH3T3 stable *KRAS* clones were transfected with three different *Vegf* promoter constructions and a plasmid containing multiple HRE inserts that were a kind gift of Dr. Pages and Dra Berra [18-21]. Construct 1 contained the complete *Vegf* Promoter, construct 2 lacked AP2 and Sp1 response elements and construct 3 lacked HRE elements. Lipofectamine 2000 was used as the transfection agent according to manufacturer’s instructions. (Invitrogen Life Technologies S.A.). At 48 h post transfection, luciferase expression was determined using the Dual Luciferase Reporter System (Promega), and the relative luciferase value was determined after normalizing to the β-Gal control. Results are expressed as mean of three independent experiments.

### Determination of DNA synthesis in HUVEC

HUVE cells (obtained from Advancell company) were deprived of growth factors for 24 h in 199 medium containing 0.1% Fetal Calf Serum. Cells were then stimulated 48 h with conditioned growth media from the different NIH3T3 clones, containing 0.25 μCi/ml (6-3H) thymidine (Amersham Pharmacia Biotech) (3 mM final concentration). After 20 hours of incubation, the incorporated radioactivity was counted by liquid scintillation. Results are expressed as a percentage of the (6-3H) thymidine incorporation in the presence of the medium of control NIH3T3.

### Ras activation assay

One mg of the protein lysate from ASP13 or CYS12 NIH3T3 cell clones was added to 30 μg of glutathione S-transferase (GST)- RBD fusion protein. RBD, encompassing amino acids 51 to 131 of Raf-1 protein, is the minimal domain required for the binding of Ras-GTP. Presence of active Ras was detected by Western blotting with monoclonal anti-pan-Ras (Oncogene Research Products mouse monoclonal clone RAS10 cat: OP40). Autoradiography bands were quantified by scanning densitometry using Quantity One Quantitation Software™ (Bio-Rad Laboratories, S.A., Alcobendas, Madrid).

### Assessment of vascular patterns

#### MicroVessel Density (MVD)

It was assessed using rat anti-PECAM (BD Pharmingene,NJ USA 550274/MEC13.3) staining on cryopreserved sections. MVD was evaluated as mean of the number of vessels in 5 hot spot fields at 400X. Only endothelial PECAM staining was considered [22].

Double immunofluorescence was used to evaluate Desmin(+)/PECAM(+). Rat anti-mouse monoclonal PEC-AM (BD Pharmingene,NJ USA 550274/MEC13.3) and rabbit anti-Desmin antibody (Lab Vision/NeoMarkers, CA, USA; RB-9014) were simultaneously incubated overnight at 4°C. Secondary goat anti-rat Alexa 546 and anti-rabbit Alexa 488 were incubated for 1 h. After washing, staining was evaluated in five hot spot fields at 400X. LEICA software counted the areas with Desmin and PECAM overlapping staining and also measured major diameters of the lumen of vessels. Correlation was performed with the presence of necrosis.

#### Immunohistochemistry

Rabbit anti α-Smooth muscle actin (Lab Vision/NeoMarkers, CA, USA; RB-9010, ready to use) staining was performed on paraffin-embedded tissue sections. Envision anti-rabbit (Dako Denmark A/S, Glostrup, DK) plus diaminobenzidine were used to visualize the reaction. Immunoreactivity was semiquantitatively evaluated by comparing the level of expression between distinct groups. Carbonic anhydrase IX (Ab15086,Abcam plc,. Cambridge, UK) and GLUT-1 (Ab652; Abcam plc. Cambridge UK) staining was also performed. Cytoplasmic positive cells were expressed as a percentage of total cells counted. For all antibodies, no staining was observed with negative control samples (absence of primary antibody, or incubation with an irrelevant antibody or IgG).

## Results

### *KRAS* codon-specific mutations induce a distinct HIF1-α and VEGF-A response

**Figure 1:**
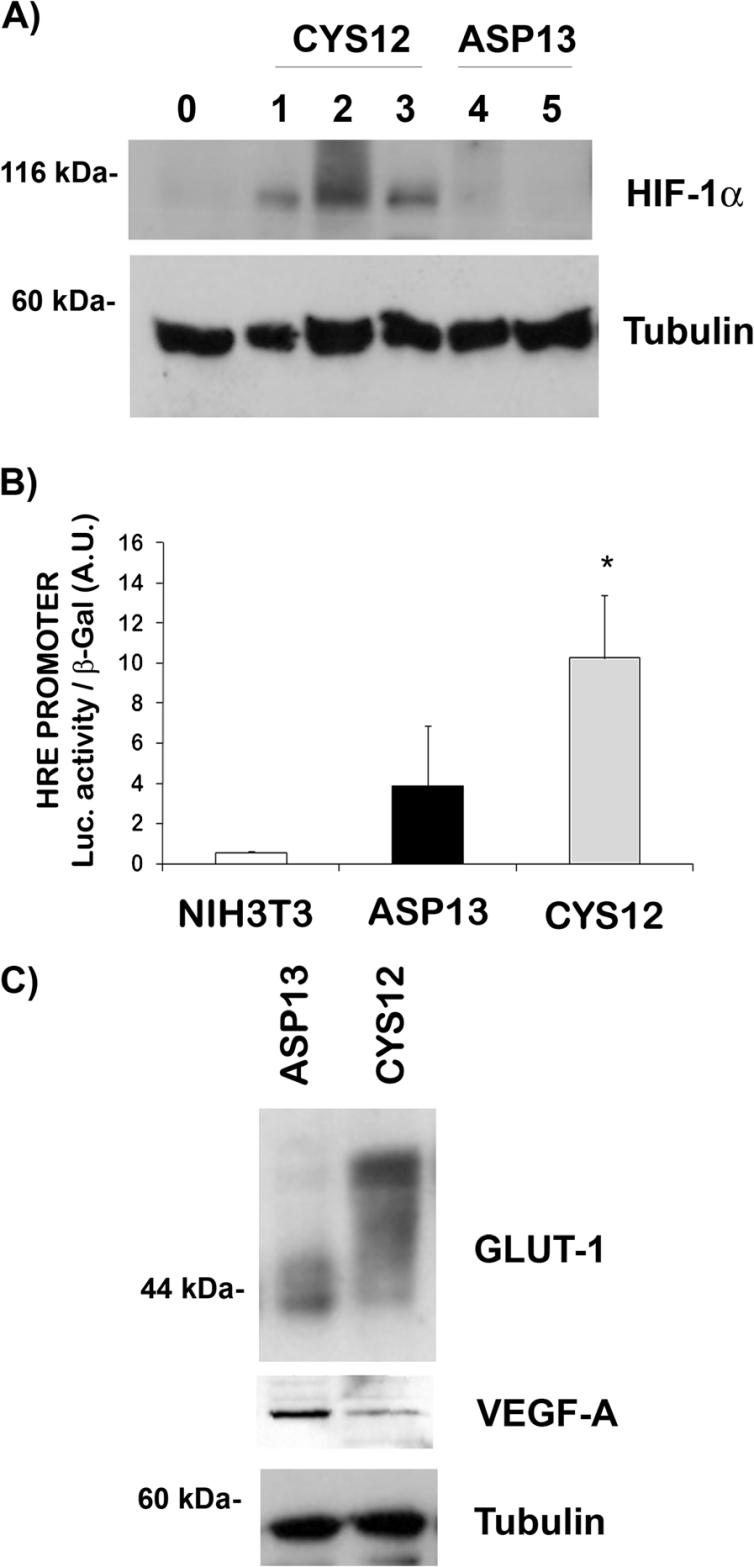
**HIF-1α, GLUT-1 and VEGF-A in cultured KRAS ASP13 and CYS12 transfectants. A)** HIF-1α protein expression was measured by western blot in NIH3T3 control cells (0), three independent clones KRAS CYS12 mutant (1, 2 and 3 show different transfected clones) and two independent clones KRAS ASP13 mutant (Clones 4 and 5). Tubulin was measured as a loading control. **B)** Luciferase activity after transfection of HIF-1α-response element promoter in NIH3T3, clone 3 KRAS CYS12 mutant and clone 4 KRAS ASP13 mutant. Results are average ± SE of three independent experiments. Activity in ASP13 mutants was significantly lower than in CYS12 mutants (P < 0.05). **C)** Protein extracts were obtained from clone 4 KRAS ASP13 mutant and from clone 3 KRAS CYS12 mutant. GLUT-1, VEGF-A and Tubulin (as a loading control) were immunodetected by western blotting. A representative blot is shown. The electrophoretic pattern of GLUT-1 depicts its glycosylation.

In normal cell culture conditions basal HIF-1α protein levels were higher in CYS12 mutants compared with ASP13 expressing cells or control NIH3T3 (Figure 1A). As expected, these basal levels of HIF-1α in the different clones analyzed increased when cells were subjected to hypoxia (data not shown). In order to confirm that HIF-1α protein was functional in our cells, we transfected NIH3T3 and NIH3T3 *KRAS* mutants cells (ASP13 or CYS12) with an extra DNA plasmid where luciferase expression was controlled by a hypoxic response element (HRE). As shown in Figure 1B, a clear correlation between HIF-1α protein levels and luciferase activity – reflecting the quantity of HIF-1α attached to the HRE [21] – existed. These findings suggest that the transcription factor was functional in normoxic cells and presented a higher activity in CYS12 *KRAS* cells.

Next, we decided to evaluate the impact of this differential expression on two HIF-1α-dependent genes, GLUT-1 the ubiquitous glucose transporter protein, and VEGF-A [9]. As observed in Figure 1C, and as expected from its more glycolytic phenotype, CYS12 mutant cells presented higher total levels of GLUT-1 as well as an increase in the glycosylated forms [23], when compared with ASP13 cells. Surprisingly, VEGF-A protein levels were higher in ASP13 cells than in CYS12 (Figure 1C).

**Figure 2:**
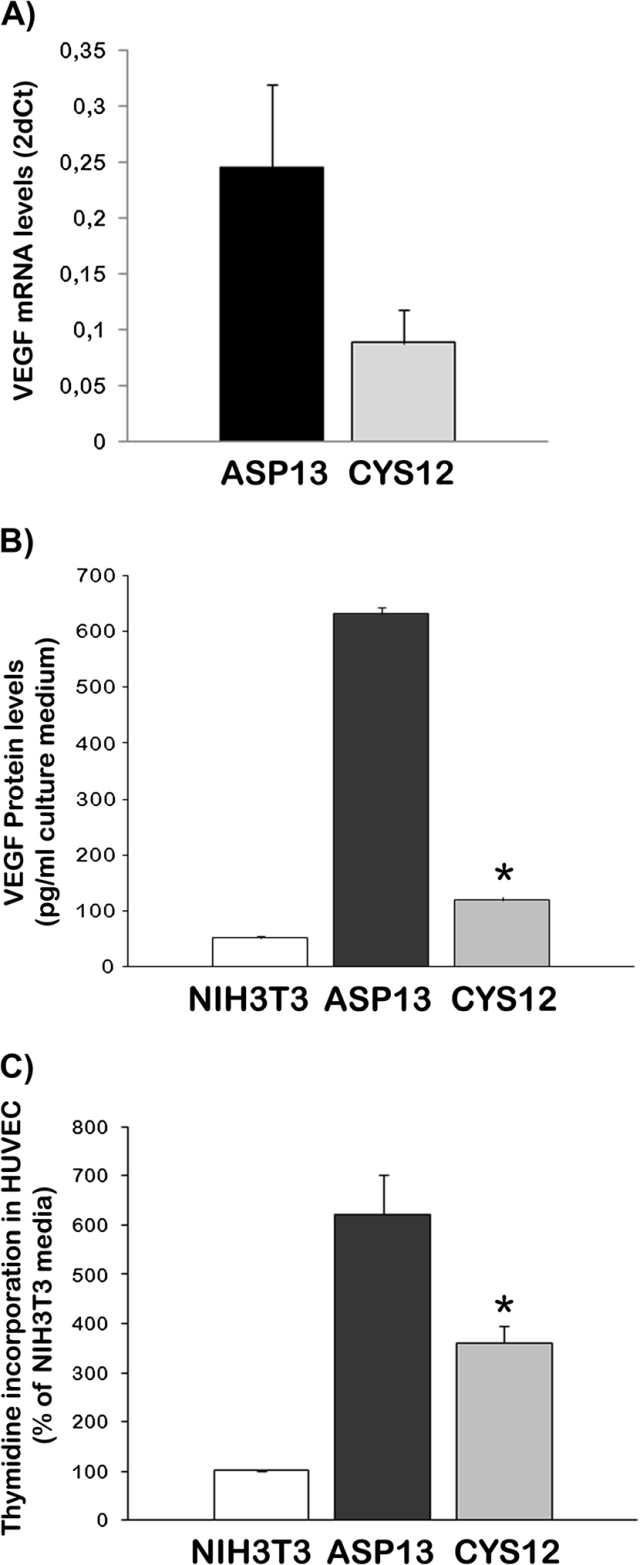
**Expression and activity of VEGF-A in KRAS transfectants. A)** VEGF-A mRNA levels were measured by RT-PCR in ASP13 or CYS12 cells. Data are expressed as average ± S.E. of 2^-ΔCT^ (ASP13: 0.468 ± 0.12; CYS12: 0.253 ± 0.08; P = 0.07) **B)** VEGF-A protein levels were measured by ELISA in conditioned media from NIH3T3 control cells, ASP13 and CYS12 transfectants (NIH3T3: 51.8 ± 3.9 pg VEGF-A165/ml medium; ASP13: 630.69 ± 12.1 pg VEGF-A165/ml medium; CYS12: 119.14 ± 6.9 pg VEGF-A165/ml medium) ASP13 cell increase five times VEGF-A 165 protein expression than CYS12. (P < 0.05). **C)** Conditioned media obtained from NIH3T3 control cells, ASP13 and CYS12 transfectants was added to HUVEC cells cultured and thymidine incorporation measured. Results are average ± S.E. of 3 independent experiments. The conditioned media from ASP13 transfectants increased HUVEC proliferation.

To confirm these differences, we analysed VEGF-A mRNA levels in our cells. A 120% increase in mRNA levels was observed in ASP13 cells compared with CYS12 transfectants (Figure 2A). Moreover, VEGF-A levels secreted in the cell culture medium were 11 times higher in ASP13 cells compared with CYS12 (Figure 2B). Finally, this VEGF-A was functional as addition of ASP13-transfectant conditioned medium to HUVEC endothelial cells resulted in higher thymidine incorporation (Figure 2C). These results suggest that *KRAS* ASP13 mutation activates a pathway that may overpass regulation of VEGF-A by HIF-1α.

### Mechanisms underlying the differential VEGF-A over expression in ASP13 cells

**Figure 3:**
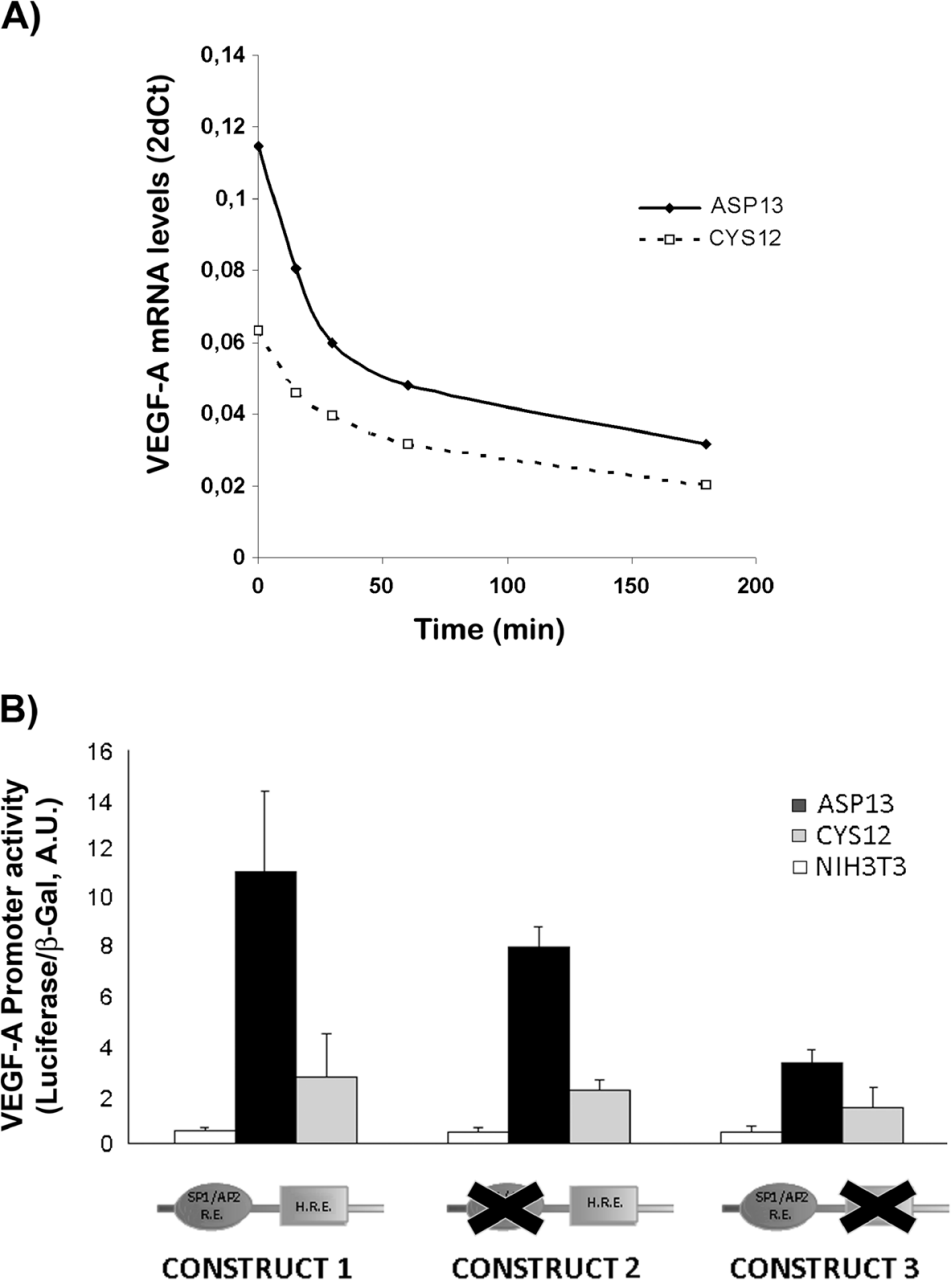
**Functional analysis of *VEGF-A *PROMOTER. A)** ASP13 or CYS12 cells were treated with actinomycin D. After the time indicated mRNA was obtained and VEGF-A mRNA levels measured by RT-PCR. Results are the average of two independent experiments. No differences in VEGF-A mRNA stability were observed in ASP13 or CYS12 cells. **B)** Luciferase activities after transfection of the complete *VEGF-A* promoter (Construct 1) or two partial deletions (Construct 2 and 3). *VEGF-A* promoter activity was increased for all constructs in Asp13 when compared to CYS12 transfectants. Promoter activity was decreased after deletion of the AP2/Sp1 response elements in ASP13 cells (P < 0.011). Results are the average ± SE of four independent experiments.

The increased amount of VEGF-A mRNA observed in ASP13 transfectants was not associated with differences in mRNA stability, measured when actinomycin D was added to the medium (Figure 3A). In contrast, activity of a construct containing the first 1176 bp of the VEGF-A promoter was 3 times higher in ASP13 cells compared to CYS12 mutated clones (P < 0.02; Figure 3B Construct 1). Together, these results indicated that differences between cells were caused by different transcriptional activities of the *VEGF-A* promoter.

Deletion of HRE within the *VEGF-A* promoter in all clones did not affect its activity. (Figure 3B Construct 2 vs Construct 1). These results further confirm the HIF-1α independent regulation of VEGF-A expression. In contrast, the selective deletion of SP1/AP2 response elements resulted in a significant decrease of *VEGF* promoter activity in both transfectants that was only significant to ASP 13 mutants (Figure 3B Construct 3 vs Construct 1).

**Figure 4:**
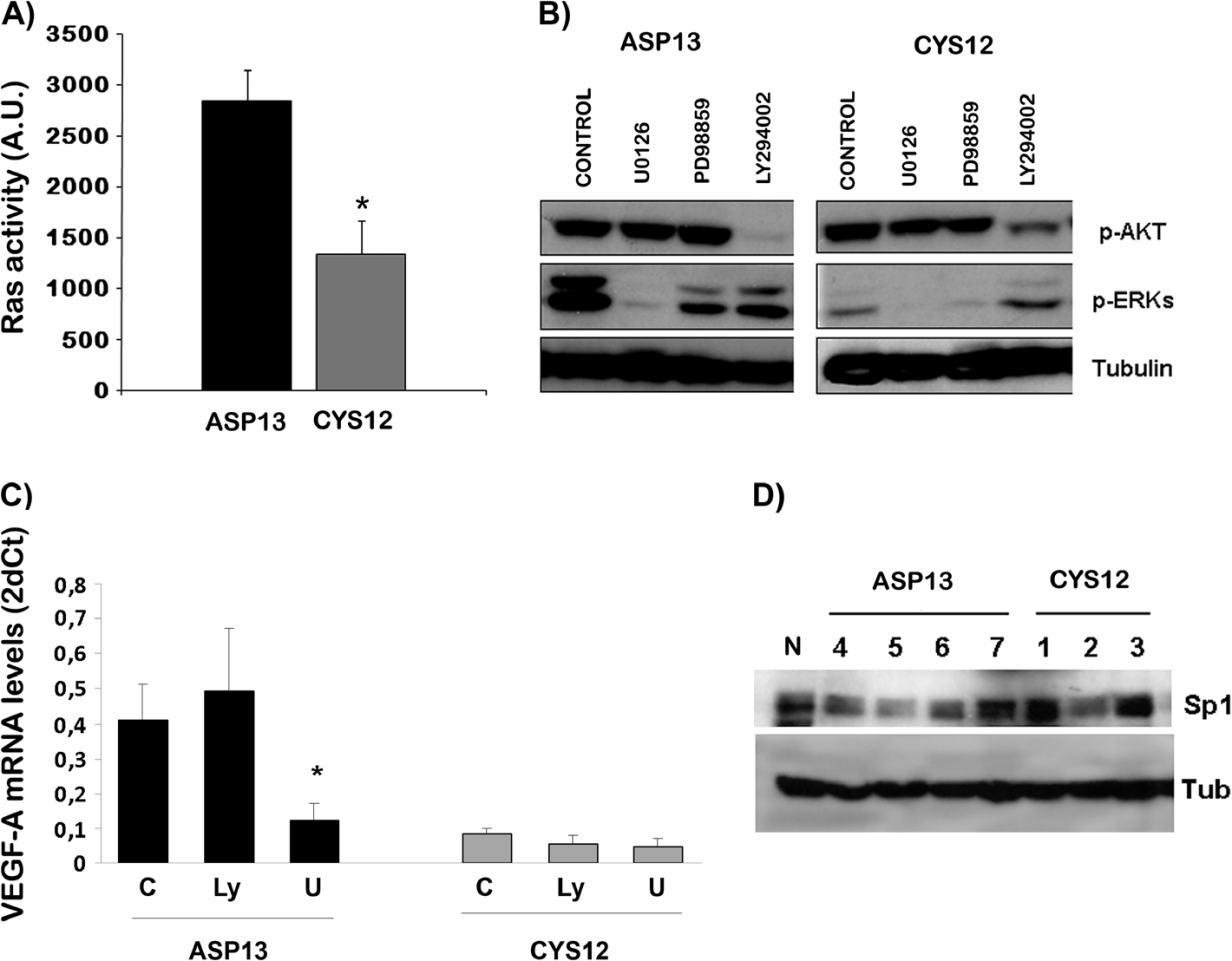
**Signalling pathway activation analysis. A)** Quantification of active Ras present in ASP13 and CYS12 cells. ASP13 transfectants showed more Ras activation (P = 0.026). **B)** Analysis of p-ERKs, p-Akt and Tubulin (as a loading control) from ASP13 and CYS12 cells treated or not with U0126 (specific inhibitor of MEK and in consequence of p-ERK) and LY294002 (specific inhibitor of PI3K and in consequence of p-Akt) is depicted. p-ERKs levels were higher in ASP13 transfectants than CYS12, while no differences were observed in p-Akt levels. Representative western blots are shown. **C)** mRNA VEGF-A levels as 2^-ΔCT^; ASP13 Control 0.40 ± 0.07; +LY294002 0.49 ± 0.1, +U0126 0.12 ± 0.04; CYS12 Control 0.08 ± 0.009, +LY294002 0.05 ±0.02, +U0126 0.046 ± 0.02. Treatment with U0126 MEK inhibitor but not with PI3K inhibitor LY294002 resulted in a reduction of mRNA VEGF-A levels in ASP13 transfectants (P = 0.031). **D)** Sp1 transcription factor levels were not different in ASP13 and CYS12 cells. Levels of Sp1 and Tubulin (as a loading control) were assayed in extracts from NIH3T3 cells (N), different ASP 13 clones (4, 5, 6 and 7) and different CYS 12 clones (1, 2 and 3).

AP2 and Sp1 are two transcription factors mainly controlled by Ras-Raf-ERKs pathway activation. [24]. In order to measure the pathway activity, we first measured Ras protein activity levels able to stimulate the ERK cascade. ASP13 clone showed an increased capacity to activate Raf (Figure 4A) that was associated with increased pERK levels (Figure 4B), while no differences were observed on PI3K cascade measured by pAKT levels. Accordingly, when ERKs activity was inhibited with U0126 for 15 minutes, a decay in mRNA VEGF-A levels was observed in ASP13 clone that was not evident in CYS12 (Figure 4C). No differences in total Sp1 protein levels were observed in mutants clones ASP13 or CYS12 (Figure 4D). In all, these results indicate that Ras-Raf-ERK-AP2/Sp1 signalling cascade is responsible for VEGF-A overexpression in ASP13 cells.

**Figure 5:**
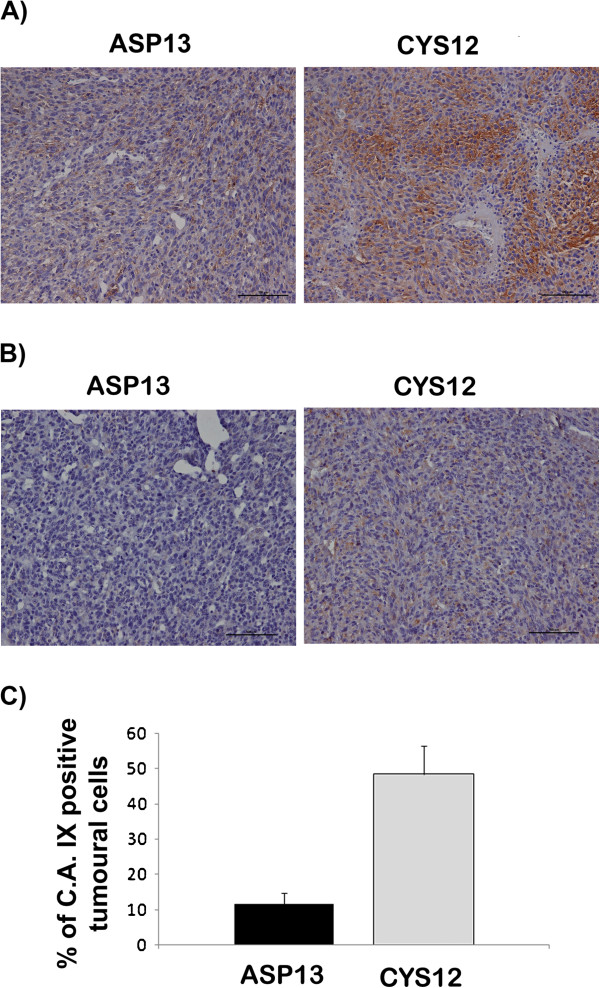
**GLUT-1 and Carbonic anhydrase IX immunostaining in KRAS ASP13 and CYS12 tumours. A)** The percentage of GLUT-1 positive cells did not differ in CYS12 and ASP13. Intensity was higher in CYS12 tumors. **B)** The percentage of Carbonic anhydrase IX positive cells and the intensity of the immunostaining is higher in CYS12 tumors. **C)** Table depicts the percentage of Carbonic anhydrase positive cells. ASP13: 11.67 ± 3.07% vs CYS12: 48.57 ± 7.9%; P < 0.002).

To study if these differences detected *in vitro* could cause a difference in the angiogenic patterns and tumoral capacity we subcutaneously injected NIH3T3 control cells and transfected clones (ASP13 or CYS12) in nude mice. In agreement with our previous observations latency period of tumors arising from distinct ASP13 transfectants was longer than for CYS12 tumors (ASP13 32.2 ± 8.9 days vs CYS12 10.4 ± 2.4; p < 0.04) (Table 1). HIF-1α activity and hypoxia was assessed though immunostaining of GLUT-1 and Carbonic Anhydrase IX. In concordance with in vitro observations, GLUT-1 immunostaining was more intense in CYS12 tumors albeit the percentage of positive cells did not among the two transfectants (Figure 5A). Differences in the expression of Carbonic Anhydrase IX were more intense, being the percentage of positive cells 4 times higher in CYS12 tumors (Figure 5B and C).

**Table 1:** Differences in latency period in vivo between ASP13 and CYS12 transfectants

	**ASP13**			**CYS12**			
	**Clone 4 (n = 10)**	**Clone 6 (n = 10)**	**Clone 2 (n = 10)**	**Clone 5 (n = 10)**	**Clone 4.3 (n = 10)**	**Clone 4.6 (n = 10)**	
**Days ± SD**	30,1 ± 6,5	24,5 ± 6,1	42 ± 0	9,7 ± 7,9	8.4 ± 0.9	13,2 ± 1,7	
**Mean ± SD**		**32.2 ± 8.9**			**10.4 ± 2.4**		**P < 0.04**

**Figure 6:**
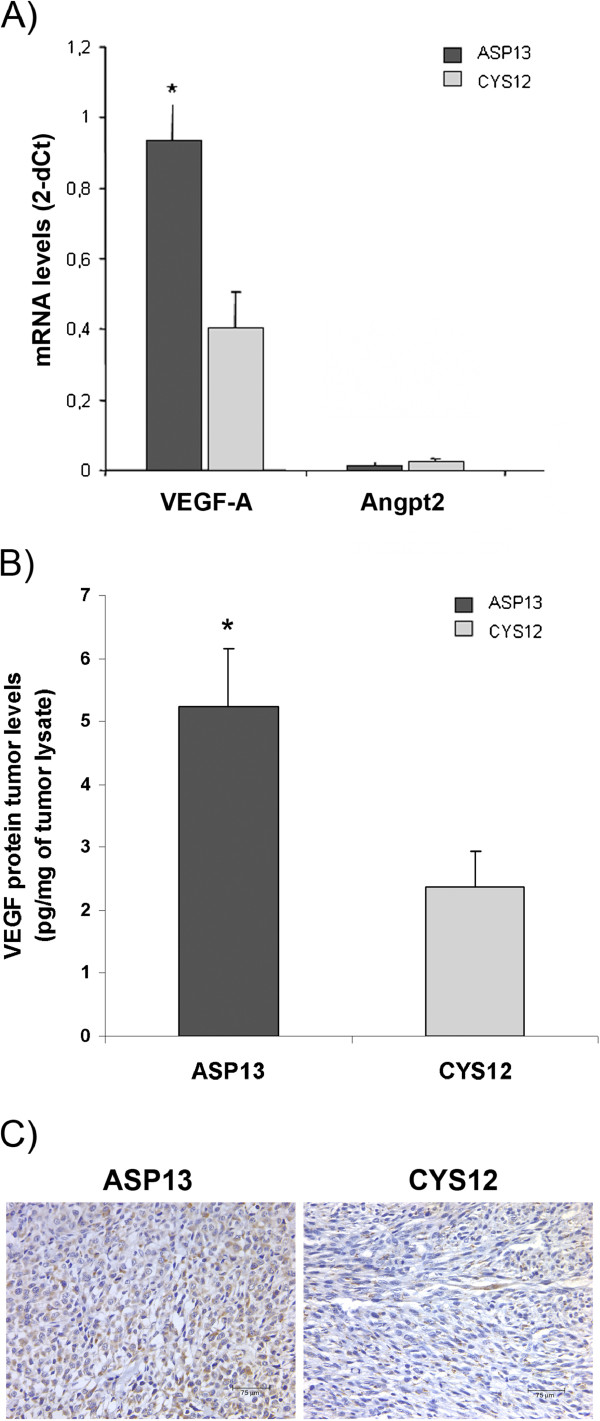
**VEGF-A expresion in KRAS ASP13 and CYS12 tumours. A)** VEGF-A mRNA and Angiopoietin-2 levels detected by real time PCR in tumour tissue. VEGF-A was overexpressed in ASP13 tumours when compared with CYS12 (ASP13: 0.9 ± 0.2 2^-ΔCT^ vs CYS12: 0.4 ± 0.1 2^-ΔCT^; P = 0.04) while mRNA Angiopoietin-2 levels were not different. **B)** VEGF-A protein levels measured by ELISA were shown as pg of VEGF per mg of tumour lysate. VEGF-A were higher in ASP13 tumours (ASP13: 5.5 ± 0.98 μg VEGF-A/mg tumour lysate vs CYS12: 2.5 ± 0.6 μg VEGF-A/mg tumour lysate; P = 0.02). **C)** Illustrative VEGF-A immunostaining is shown for each mutant.

We confirmed that mRNA VEGF-A levels were also higher in ASP13 tumours compared with CYS12 (Figure 6A). The same trend was observed at the protein VEGF-A level, as assessed by ELISA and immunostaining (Figure 6B and C). In contrast, angiogenic factor Angiopoietin 2 levels did not show differences between tumours (Figure 6A).

#### Tumor growth vascular patterns

**Figure 7:**
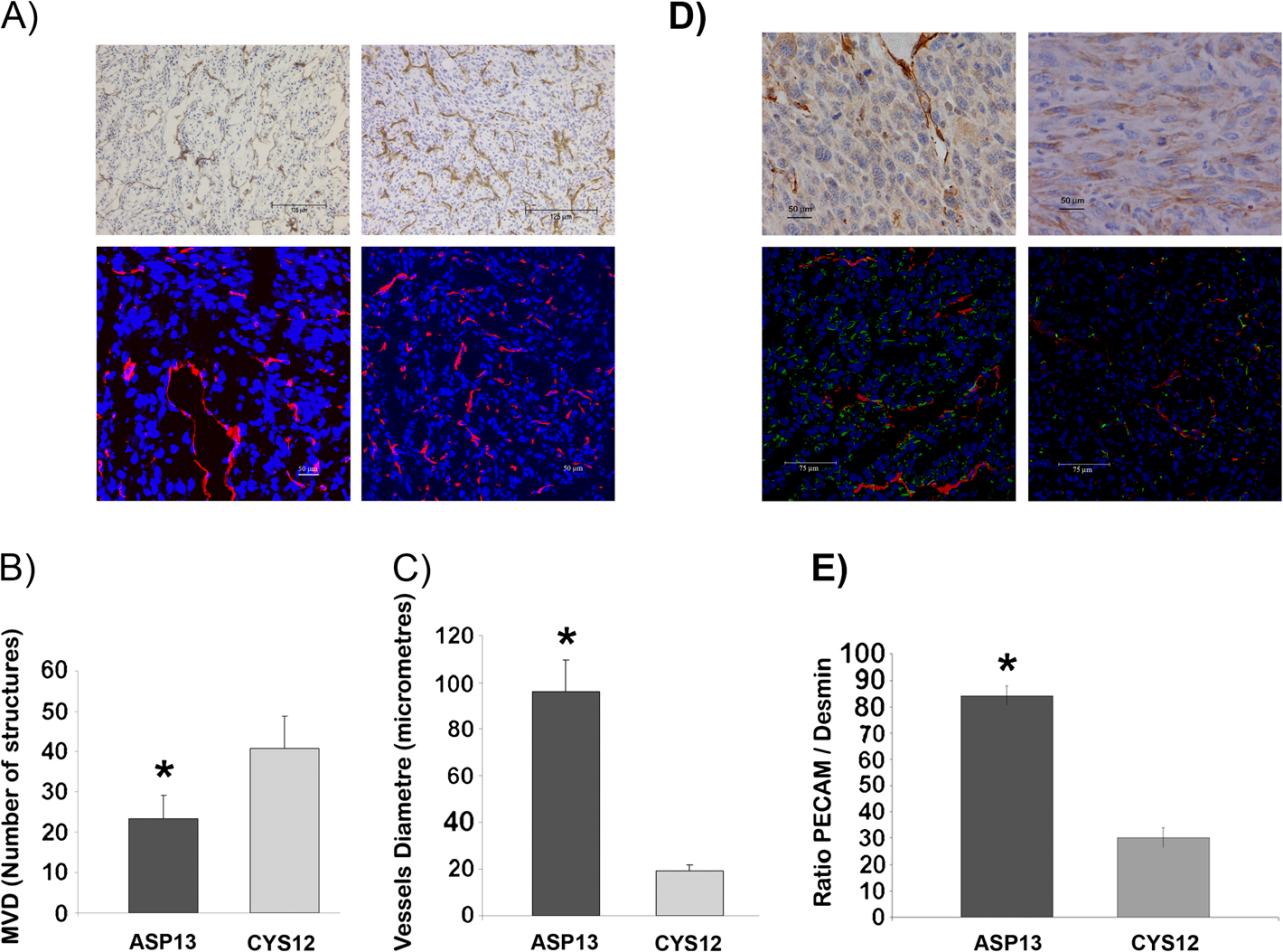
**Vessel number in ASP13 and CYS12 subcutaneous tumours. A)** PECAM immunohistochemistry (up) and PECAM immunofluorescence (bottom) staining of subcutaneous tumours are shown as a representative example of each tumour mutation group. (ASP13 on the left and CYS12 on the right). **B)** Microvessel density (MVD) was represented as number of vessels structures PECAM+/microfield at 400X. (CYS12 40.8 ± 8 vs ASP13 23.7 ± 6 number of PECAM (+) vessels; P = 0.002). **C)** Mean of vessel diameter is shown in each bar. (ASP13: 96,4 ±12,9 μm vs CYS12: 19.3 ± 2.8 μm; P < 0.0001). **D)** Representative staining of ASP13 (left) and CYS12 (right) tumour tissues are shown. Up: αSMA of paraffin tumour tissue. Bottom: PECAM/Desmin staining on cryopreserved tumour tissue. Positive mural cells were more evident in ASP13 tumours. **E**) PECAM/Desmin ratio analysed by LEICA software was represented. Ratio was higher in ASP13 tumours (P < 0.0001).

The distinct VEGF-A production observed was associated with a specific vascular pattern. On the one hand, vascular hotspots zones with distended vessels were apparent in ASP13 tumours, with generation of haemorragic and necrosis zones (Figure 7A). On the other hand, microvessel density (MVD) was higher in CYS12 (Figure 7B), being the diameter of vessels higher in ASP13 tumours (Figure 7C). Finally, vessels from ASP13 tumours were surrounded by mural cells that stained positive for α-Smooth Muscle Actin and Desmin proteins, while mural cells were scarce around CYS12 tortuous vessels (Figure 7D and E). These different vascular patterns do not associate with significant differences in the degree of necrosis among the two transfectants.

## Discussion

In the context of *KRAS-*driven tumourigenesis, mutations located at codon 12 and 13 display distinct malignant potential and differentially regulate apoptosis, cell cycle [4,15], or metabolic profiles [25]. Here we show that minor differences in the molecular nature of *KRAS* mutations stimulate distinct intracellular signalling pathways in normoxic conditions with different impact in basal levels of HIF-1α VEGF-A production and generation of a distinct vascular network in tumours.

Upregulation of VEGF by the KRAS pathway has been previously shown [26]. Here we show that cells expressing ASP13 *KRAS* mutant present higher levels of VEGF-A, the main pro-angiogenic gene induced by hypoxia, in the absence of high HIF-1α levels [9]. In contrast, CYS12 mutants present a high glycolytic phenotype [25] through HIF-1α-dependent induction of glycolytic enzymes including GLUT-1 glucose transporter supporting the role of HIF-1α in switching to a glycolytic metabolism [7].

We have attempted to gain insight into the molecular mechanisms underlying the differential VEGF-A overexpression, apparently independent of HIF-1α in ASP13 clones, Our data support a direct transcriptional effect of ASP13 acting on *VEGF-A* promoter. This effect is mediated by a distinct activation of Raf-ERKs pathway and AP2/Sp1 elements within the proximal *VEGF-A* promoter. Of note it is independent of hypoxia-dependent elements and of PI3K activity. Extracellular signals that induce VEGF-A through this proximal region include, among others, growth factors such as EGF, insulin and PDGF in fibroblasts [18], prostaglandin E2 in human muscle cells [27], M-CSF in monocytes [28] and lysophosphatidic acid (LPA) in ovarian cancer cells [29]. All of them affect promoter activity through modulation of at least Sp1 transcriptional activity. Noteworthy, Sp1 is also regulated by different signalling pathways including ERKs, PKA and PI3K-Akt [18,30]. We have not detected changes in total Sp1 protein levels between ASP13 and CYS12 mutants, but other mechanisms with impact in the activity of this transcription factor could be implicated, such as acetylation, sumoylation, glycosylation or phosphorylation [24].

In our xenograft model, ASP13 xenografts consistently develop angiogenic sprouts of large diameter, invested by mural cells. These structures seem to be sufficient to support the increased utilization of the oxidative pentose phosphate pathway observed in the more benign ASP13 tumours [25]. While development of these complex vascular structures may account for the initial delay observed in tumour growth, we speculate that they are able to support the very rapid growth occurring later [4]. Nonetheless, the presence of significant tumour necrosis and less Carbonic anhydrase IX to hypoxic adaptation, observed in established ASP13 tumours may depict the relative insufficiency of this vascular tree [8]. In contrast, histological analysis reveals that the more aggressive CYS12 tumours educe a dense endothelial-lined microvascular network that allows an early, steady and sustained tumour growth. This vascular strategy appears to be effective for these tumour cells that are more resistant to hypoxia, do not proliferate fast [31] and have relatively low energetic requirements associated with an increased anaerobic glycolysis.

The vascular pattern observed in ASP13 xenografts is in line with previous observations linking high VEGF-A levels with an increased diameter of newly forming vessels [32,33]. The prominent stimulation of DNA synthesis in primary HUVECs by whole ASP13 conditioned medium, and in a less conspicuous manner by CYS12 supernatants, propose significant paracrine effects of tumour cell-derived VEGF-A in neovascularization [34]. Also, ASP13 tumours vessels are covered with α-Sma(+)/Desmin(+) cells [35] further highlighting the contribution of VEGF-A to vessel maturation and tumour growth.

The retarded growth of ASP13 tumours harbouring elevated VEGF-A levels is consistent with reports challenging the concept that VEGF is just a positive angiogenic regulator. While angiopoietin2 levels did not show differences between transfectants, we cannot exclude a role of other angiogenic factors in differences observed between ASP13 and CYS12 tumoral vessels [36].

The impact of the genetic background of tumour cells on the angiogenic phenotype is relevant since they may have consequences regarding efficacy of specific antiangiogenic strategies. An evolving tumour with an ever-changing genetic background likely educes a dynamic vascular strategy that may escape to specific antiangiogenic treatment such as those targeting VEGFRs or its ligand [37]. This is of importance now that more antiangiogenic drugs are being introduced to the clinical setting and there is a need for biomarkers that help in the selection of patients to be treated. *KRAS* mutations are used as negative predictors of antiEGFR therapies in colorectal cancer [38]. The role of *KRAS* mutation as a predictive marker of bevacizumab-based treatment has been also explored. Indeed, better response rates to bevacizumab can be observed in *KRAS* wt colorectal tumors when compared to *KRAS* mutant [39]. Of note, some authors have explored a potential differential behaviour of codon 13 mutant tumors with no conclusive results [40]. It will of interest to explore in the adequate clinical setting whether our experimental observations correlate with clinical outcome in other tumor types such as colorectal cancer.

## Conclusions

Mutations in the *KRAS* gene are among of the most prevalent in human tumours and they are known to have pleiotropic effects on tumour biology. The less aggressive ASP13 mutation, through Raf-Ras-ERKs activation of the VEGF-A promoter, creates a prominent VEGF-A-associated vascular network in the absence of high HIF-1α levels. This vascularisation is less effective than the dense microvascular network observed in CYS12 tumours. In our model system, we have shown that the molecular nature of *KRAS* mutations clearly influences the vascular strategy devised by the tumour cell. These observations provide us with a deeper insight of the complex role of major angiogenic regulators such as VEGF on tumour vasculature development and their relationship with oncogene activation.

## Competing interest

None of the authors have any financial or non-financial competing interests in relation to this paper.

## Authors’ contributions

AF: study concept and design; acquisition of data; analysis and interpretation of data; drafting of the manuscript; critical revision of the manuscript. MAA: study concept and design; acquisition of data; analysis and interpretation of data; drafting of the manuscript; critical revision of the manuscript; MTQ: acquisition of data; analysis and interpretation of data; critical revision of the manuscript; FV: study concept and design; acquisition, analysis and interpretation of data; drafting of the manuscript; critical revision of the manuscript; obtained funding; study supervision; JRG: material support; obtained funding; critical revision of the manuscript; GC: study concept and design; analysis and interpretation of data; drafting of the manuscript; critical revision of the manuscript; obtained funding; study supervision. All authors read and approved the final manuscript.

## Pre-publication history

The pre-publication history for this paper can be accessed here:

http://www.biomedcentral.com/1471-2407/13/125/prepub
